# BDNF-induced BDNF release: A virtuous loop for the cardioprotective effects of exercise in post-ischemic heart failure

**DOI:** 10.1016/j.ijcha.2025.101623

**Published:** 2025-01-24

**Authors:** Abdulbaset Maroofi, Fatemeh Safari, Asghar Abbasi

**Affiliations:** a Department of Exercise Physiology University of Guilan Rasht Iran; b Stem Cell Biology Research Center Yazd Reproductive Sciences Institute Shahid Sadoughi University of Medical Sciences Yazd Iran; c Department of Physiology Faculty of Medicine Shahid Sadoughi University of Medical Sciences Yazd Iran; d Division of Respiratory & Critical Care Physiology & Medicine. The Lundquist Institute for Biomedical Innovation at Harbor-UCLA Medical Center CA USA

Dear Editor

Over the last few decades, therapeutic advancements have markedly reduced premature mortality following myocardial infarction (MI). However, post-MI deleterious molecular and cellular alterations, coupled with the loss of a copious number of cardiomyocytes, set up the stage for the development of heart failure (HF). This chronic and profound myocardial dysfunction can manifestly jeopardize the everyday life of survivors. Thus, targeting the key molecular players governing post-MI cardiac function, particularly through non-pharmacological interventions, is paramount to overthrowing HF.

Brain-derived neurotrophic factor (BDNF), a well-recognized pleiotropic neuroprotective molecule, is undisputedly vital for the cardiovascular system. Recent investigations confirm a significant correlation between lower circulating levels of BDNF and worse clinical outcomes in HF patients [1]. Similarly, low serum BDNF levels have been shown to predict poor response to cardiac rehabilitation programs in patients with cardiovascular disease [2]. In addition, there is unequivocal evidence demonstrating that cardiac BDNF and its sarcolemmal receptor, tropomyosin-related receptor kinase B (TrkB), are required for optimal myocardial contraction and relaxation [3]. In the myocardium, through binding to the TrkB receptor, endogenous BDNF triggers a Ca^2+^/calmodulin-dependent protein kinase II (CaMKII) signaling cascade, thereby modulating myocardial mechanical performance. For instance, administration of BDNF to healthy rodents augments myocytes' sarcomere shortening and Ca2 + cycling, whereas deleting sarcolemmal TrkB obviously impairs basal myocardial contraction and relaxation [3]. Emerging evidence suggests that the ablation of cardiomyocyte-derived BDNF during embryonic development does not affect survival rates or growth; however, in young adult hearts (3-month-old mice), it results in myocardial erosion and heart failure pathology, characterized by cardiomyocyte death, fibrosis, hypertrophy, inflammation, reactive oxygen species (ROS) overactivity, and cardiac dysfunction. The ablation during the developmental phase also caused the exacerbation of post-MI cardiac dysfunction and impinged on the regeneration process in adult hearts [4]. One study showed that endogenous myocardial BDNF expression, and, thus, TrkB activation, alleviates oxidative stress and apoptosis in septic myocardial dysfunction via stimulation of the eNOS/NO pathway [5]. Another recent study [6] demonstrated that chronic MI depletes cardiac BDNF content, while TrkB agonists arrest HF by promoting angiogenesis and boosting cardiomyocyte function. This study also found that sustained activation of TrkB cultivates autologous BDNF in the myocardium of mice with ischemia/reperfusion (I/R) injury, protecting cardiac function. Intriguingly, further assessments manifested that superfusion of cardiomyocytes with a β3AR-agonist triggers local BDNF generation and, by doing so, abrogates post-MI repercussions in the heart. These experiments ultimately confirmed a new cardioprotective loop with promising potential for mitigating ischemic injury. The process initiates with circulating BDNF, which stimulates the cardiac sarcolemmal TrkB, leading to increased BDNF production within cardiomyocytes, which in turn reactivates the TrkB, perpetuating the beneficial cycle.

Exercise, particularly aerobic exercise training, is well established to strengthen the heart and reduce the risk of coronary heart disease. It elicits profound positive effects on molecular signaling pathways, simultaneously inhibiting harmful mechanisms and activating protective ones, providing dual cardioprotective benefits [7]. During exercise bout(s), peripheral tissues, specifically skeletal muscles, release various exercise-induced factors (exerkines) into the circulation, including lactate, irisin, interleukin-6 (IL-6), cathepsin B (CTSB), 3-hydroxybutyrate (3OHB), and endocannabinoids. These molecules pass through the physical barriers (e.g., blood brain barrier) and communicate directly to the receiving organs/tissues, resulting in activation of different signaling pathways [8]. BDNF is one of the beneficial exercise-induced factors that can be released into the circulation by both acute and chronic exercises. Either at rest or in response to exercise, the brain is mainly responsible for releasing BDNF into the circulation [8]. Exercise can also directly activate the BDNF/TrkB signaling in the heart. Notably, exercise training has been shown to restore cardiac function in post-MI rats by activating myocardial BDNF/TrkB signaling and its key downstream effectors, CaMKII and Akt [9]. Similarly, exercise training in post-MI rats has been shown to enhance angiogenesis and improve cardiac function by amplifying BDNF/TrkB signaling in the myocardium [10]. Recently, we demonstrated that intense treadmill running for 12 weeks increases cardiac BDNF protein expression levels not only in healthy rats but also in Western diet-induced inflammatory cardiomyopathy, which was accompanied by improved cardiac function [11]. Other investigators have also showed that exercise, as opposed to HF, fortifies cardiac BDNF levels, enhancing myocardial bioenergetics in mice [12]. Equally important, inhibition of cardiac TrkB has been shown to destroy the actual impacts of exercise on cardiac function, vascularization, and cellular bioenergetics [9], [10], [12]. The BDNF system has also been labeled as a target through which exercise can provide a robust protection for the heart-brain axis in patients with HF [13]. Mechanistically, exercise seems to rely on multiple routes to activate the BDNF/TrkB signaling in the heart. In particular, cardiomyocyte sarcolemmal TrkBs tend to sequester circulating BDNF molecules released by exercise, thereby stimulating BDNF generation within the heart. BDNF produced by cardiomyocytes, in turn, stimulates TrkB in an autocrine fashion, leading to further release of BDNF. In other words, exercise creates a feedback loop where circulating BDNF and cardiac BDNF stimulate the release of more BDNF from cardiomyocytes, a process that can be suggested as *“BDNF-induced BDNF release”*. Similar to skeletal muscle, where contractions induce BDNF expression, increased myocardial contraction or augmented sympathetic outflow to the heart during exercise may trigger BDNF release from cardiomyocytes per se. From this perspective, it is very plausible that exercise evokes the expression of cardiac BDNF by recruiting β-adrenergic receptors, particularly the β3AR type. Further, knowing that overexpression of sirtuin 1 (SIRTI) leads to the upregulation of cardiac BDNF [14], exercise may contribute to BDNF production by targeting cardiac SIRT1. Beyond these, by boosting laminar shear stress, exercise might open the way for activation of the BDNF/TrkB signaling and stimulation of myocardial angiogenesis in a nitric oxide (NO) −dependent manner [10]. Finally, since irisin can regulate the expression of BDNF [15], exercise may positively affect cardiac BDNF levels by augmenting irisin, both in circulation and cardiomyocytes (Fig. 1).

**Fig. 1:**
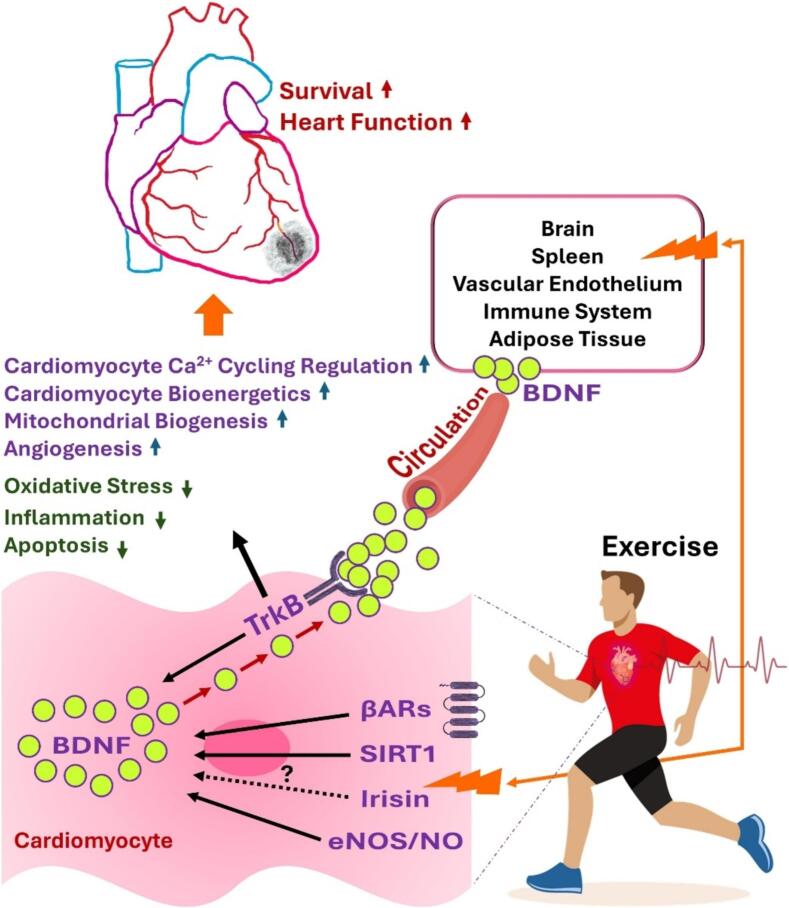
Cardioprotective mechanisms of exercise through the activation of brain-derived neurotrophic factor (BDNF)/tropomyosin receptor kinase B (TrkB) signaling. β-ARs, beta-adrenergic receptors; SIRT1, sirtuin 1; eNOS, endothelial nitric oxide (NO) synthase.

In conclusion, this letter aims to elucidate the mechanisms by which the BDNF-TrkB loop is activated by exercise, which may support proper heart function and potentially reduce the risk of HF following MI. Aerobic exercise training appears to modulate multiple molecular pathways, contributing to the components of a novel and promising cardioprotective mechanism. Nevertheless, it is important to note that these findings are primarily derived from preclinical studies, and the integrated protective effects observed may not translate uniformly to human patients. Despite this limitation, the insights provided may pave the way for novel prognostic and therapeutic strategies.

## Declaration of competing interest

The authors declare that they have no known competing financial interests or personal relationships that could have appeared to influence the work reported in this paper.
